# Supplementary material for: Quantization of events in the event-universe and the emergence of quantum mechanics
Source: Sci Rep. 2023 Oct 19;13:17865. doi: 10.1038/s41598-023-44550-4 (PMC10587342; doi:10.1038/s41598-023-44550-4)
Supplement: Supplementary file 1 — Supplementary Information. [file 41598_2023_44550_MOESM1_ESM.pdf]

Appendix. Quantum foundations.

The Bohmian mechanics approach to quantum theory (that is the improvement of De Broglie double solution theory) is still not commonly accepted in the physical community. Therefore it is useful to say a few words about Bohmian mechanics by highlighting the issues related to our paper. The mathematical formalism of Bohmian mechanics is closer to classical mechanics than the standard complex Hilbert space formalism. One of the basic equations of this model is the Hamilton-Jacoby equation with the additional potential, known as the quantum potential. The latter is nonlocal and this is the source of nonlocality of Bohmian mechanics. Another basic equation is the continuity equation. The phase plays the role of action. Bohmian mechanics is well accommodated with curved space, since all quantities, as say gradient can be in use [40]. Once again we stress that experimentally the Bohmian and complex Hilbert space model cannot be distinguished. Numerous attempts to design an experimental setup to distinguish these models were not successful.

This is the good point to remark that sometimes quantum physics is identified with one concrete mathematical model, namely, the complex Hilbert space formalism with the mathematical description of states as normalized vectors (or more generally density operators) and observables as Hermitian operators (or more generally POVMs). However, this is not correct approach. A few totally different formalisms can be used for the same physical theory. Besides Bohmian mechanics, we can mention the algebraic approach that is popular in quantum field theory. We can also mention Feynman's path integral formalism.

In this paper we follow the Bohr's interpretation of QM, sometimes called the Copenhagen interpretation: QM is a theory of measurements. It is not about quantum reality as it is, it is about outputs of measurements. The latter are performed by classical macroscopic apparatuses. So, quantumness is in data, at the epistemic level. Therefore, Bohr pointed out that quantum formalism, especially the complementarity principle, can be used outside of physics, especially in biology, for modern realization of this idea see the review [41]. Further more we would like to refer the reader to Penrose et al. quantum theory of cognition [42].

We also note that QM is characterized by the diversity of interpretations. We also remark that even the term "Copenhagen interpretation" is characterized by the diversity of meaning assigned to it.
